# Lung Cancer Screening in Asbestos-Exposed Populations

**DOI:** 10.3390/ijerph19052688

**Published:** 2022-02-25

**Authors:** Steven B. Markowitz

**Affiliations:** Barry Commoner Center for Health and the Environment, City University of New York, New York, NY 11376, USA; smarkowitz@qc.cuny.edu

**Keywords:** asbestos, lung cancer, screening, surveillance, low dose CT scan, occupation, exposure

## Abstract

Asbestos exposure is the most important cause of occupational lung cancer mortality. Two large randomized clinical trials in the U.S. and Europe conclusively demonstrate that annual low-dose chest CT (LDCT) scan screening reduces lung cancer mortality. Age and smoking are the chief risk factors tested in LDCT studies, but numerous risk prediction models that incorporate additional lung cancer risk factors have shown excellent performance. The studies of LDCT in asbestos-exposed populations shows favorable results but are variable in design and limited in size and generalizability. Outstanding questions include how to: (1) identify workers appropriate for screening, (2) organize screening programs, (3) inform and motivate people to screen, and (4) incorporate asbestos exposure into LDCT decision-making in clinical practice. Conclusion: Screening workers aged ≥50 years with a history of ≥5 years asbestos exposure (or fewer years given intense exposure) in combination with either (a) a history of smoking at least 10 pack-years with no limit on time since quitting, or (b) a history of asbestos-related fibrosis, chronic lung disease, family history of lung cancer, personal history of cancer, or exposure to multiple workplace lung carcinogens is a reasonable approach to LDCT eligibility, given current knowledge. The promotion of LDCT-based screening among asbestos-exposed workers is an urgent priority.

## 1. Introduction 

Critical elements of the rationale in support of lung cancer screening among asbestos-exposed populations have been demonstrated and include:

(1) The significant burden of continuing lung cancer incidence and mortality imposed by asbestos exposure;
(2) The globally accepted scientific consensus that low-dose CT (LDCT) scan screening reduces lung cancer mortality; and
(3) Increasing evidence that the optimal approach to identifying populations who are likely to benefit from lung cancer screening addresses a broad range of lung cancer risk factors, in addition to age and smoking;
(4) The application of low-dose CT scan-based screening to worker populations exposed to asbestos and other occupational lung carcinogens has demonstrated success in acceptance of screening and the detection of lung cancer at early stages.

Empiric evidence that is specific to asbestos-exposed populations (AEPs) that the application of low dose CT scanning is effective at early lung cancer detection and/or mortality reduction is limited, as will be reviewed. There have been, for example, no randomized clinical trials of low dose chest CT scanning among AEPs. Importantly, given the results of two decades of research on the use of the low dose chest CT to reduce lung cancer mortality, randomized clinical trials specific to AEPs are neither feasible nor likely to be undertaken and would raise important ethical questions.

Current challenges to the successful application of lung cancer screening for AEPs are substantial but are highly amenable to progress. They include:

(1) Establishing a consensus about the characteristics of asbestos exposure in association with other lung cancer risk factors that determine likely benefit from and suitability for lung cancer screening;
(2) Developing and testing methods to identify and enroll at risk AEPs in LDCT screening;
(3) Modifying health care information systems and health provider practice to facilitate identification and inclusion of asbestos-exposed populations in LDCT screening; and
(4) Educating and motivating asbestos-exposed populations to participate in annual LDCT-based screening.

These challenges exist for other occupational lung carcinogens, such as silica, diesel exhaust, welding, selected metal exposures, and others, so that overcoming such obstacles for AEPs will permit the generalization of lessons learned on AEPs to other workers at risk for occupational lung cancer [1].

## 2. Current Knowledge

### 2.1. Burden of Asbestos-Related Lung Cancer

Although the ban or severe restriction on the new use of asbestos-containing products in at least 67 countries has diminished continued exposure to asbestos [2], the incidence of cancers due to prior asbestos use in these same countries has abated much more slowly, if at all [3]. Current asbestos-associated diseases derive from exposures that occurred decades ago and predictably appear in the absence of any effective chemoprevention measures after the requisite multi-decade latency period. Asbestos-related diseases as a whole, whether cancer or fibrosis, remain largely resistant to treatment, so that asbestos-related mortality remains substantial well into the 21st century. Additionally, unfortunately, asbestos-related diseases and associated mortality will continue for decades into the future in view of the continued large scale continued use of asbestos in many countries, including China, Russia, India, and others.

Lung cancer remains the most common cause of cancer mortality in the world, causing nearly one in five (20.4%) cancer deaths in 2019 [4]. A substantial proportion of all lung cancers, in the range of 10% to 15%, are caused collectively by the more than two dozen occupational exposures that are recognized lung carcinogens in humans [1,5,6,7].

Asbestos is the single most important cause of occupational lung cancer mortality in the world. It accounted for an estimated 30% of occupational lung cancer deaths globally in 2015 [8], and substantially higher proportions in some countries: 41% of occupational lung cancer deaths in Great Britain [9], and 46% of occupational lung cancer cases in Canada [10]. Other common and important occupational lung carcinogens include silica, diesel exhaust, nickel, cadmium, chromium, and welding [1,11].

Estimated numbers of lung cancer deaths globally per year attributed to asbestos range from ~55,000 to ~302,000 with a point estimate of ~184,000 [12]. The magnitude of lung cancer deaths due to asbestos is clearly substantial. Ironically, among asbestos-related cancers, malignant mesothelioma often attracts more attention than lung cancer due to the former cancer’s specific link to asbestos, but lung cancer due to asbestos is at least twice as common as malignant mesothelioma [12,13].

### 2.2. LDCT Reduces Lung Cancer Mortality

Two large complementary randomized clinical trials of populations at high risk of lung cancer—the U.S. National Lung Screening Trial (NLST) and the Dutch–Belgian Nederlands–Leuvens Longkanker Screenings Onderzoek (NELSON)—conclusively demonstrated that periodic low dose chest CT scanning reduces lung cancer mortality [14,15].

The NLST, conducted by the United States National Cancer Institute, included 53,454 enrollees aged 55 to 74 who had smoked at least 30 pack-years and, for former smokers, had quit within the past 15 years. The CT versus chest X-ray study arms were screened annually for 5 years and followed for a median of 6.5 years. The CT screen arm showed a 20% reduction in lung cancer mortality versus the CXR screen arm [14]. The NELSON trial included 13,195 men and 2594 women aged 50 to 74 years who had smoked at least 15 to 20 pack-years and, if former smokers, had quit 10 of fewer years prior to the study. NELSON compared a group who underwent 4 rounds of CT screening (baseline, year 1, year 3, and year 5.5) with a group who had no screening; all were followed for at least 10 years. NELSON observed a 24% and 33% lung cancer mortality reduction among men and women, respectively, in the trial [15].

Based initially on the NLST with a subsequent revision following publication of results of NELSON and modeling studies, the United States Preventive Service Task Force (USPSTF) issued two sets of recommendations on LDCT screening eligibility. In 2013, the USPSTF recommended annual low dose CT scanning for high-risk individuals, who were defined as people aged 55 to 74 years who had smoked at least 30 pack-years of cigarettes and currently smoke or quit less than 15 years previously. These recommendations were subsequently adopted by the U.S. Federal government and private insurance companies for use in health insurance coverage and clinical preventive practice. In 2021, the USPSTF revised its recommendations for eligibility to include people aged 50 to 80 years who have at least a 20 pack-year smoking history and currently smoke or have quit within the past 15 years [16].

A group of European physicians and scientists who are leaders in lung cancer screening in Europe have developed a set of consensus recommendations on lung cancer screening, which were published in June 2020 [17]. They recommended the use of lung cancer risk thresholds (e.g., 1.51% over a 6 year period) applied to results of risk prediction models in order to determine who should be eligible for LDCT-based lung cancer screening. Of the numerous risk prediction models that have been developed, the expert group favored those derived from the Liverpool Lung Project (LLP_v2_, developed in England) and the Prostate, Lung, Colorectal and Ovarian Cancer Screening Trial (PLCO_m2012_, developed in North America).

Important research issues remain beyond the critical ones of LDCT efficacy and eligibility. They include, at a minimum, optimizing LDCT and risk factor information to tailor the application of screening; cost-effectiveness; the integration of biomarkers to improve risk stratification and cancer identification; identifying the best screening intervals; and how best to implement LDCT screening in target populations [18]. 

### 2.3. Basing Eligibility on Age and Smoking Alone Versus a Broader Set of Lung Cancer Risk Factors

The NLST and NELSON clinical trials and all the smaller clinical trials [19] used age and smoking as the sole risk factors for lung cancer to be eligible for participation in the trials (NLST: 55 to 74 years; ≥30 pack-years; and ≤15 years since smoking cessation) (NELSON: 55 to 75 years; >15 cigarettes per day for >25 years or >10 cigarettes per day for >30 years; ≤10 years since smoking cessation). Limited data on other risk factors were collected, though they were not examined in the main study analyses. The NLST collected data on asbestos exposure, but ~5% of participants reported a history of asbestos exposure, precluding a useful analysis.

More than 20 lung cancer risk prediction models based on large lung cancer datasets (e.g., Prostate, Lung, Colorectal and Ovarian Cancer Screening Trial (PLCO); Liverpool Lung Project (LLP)) have been developed, and many are inclusive of a broader and more detailed set of lung cancer risk factors than the NLST and NELSON trials. These additional risk factors variably include gender, race, body mass index (BMI), intensity and duration of smoking, number of years since smoking cessation, chronic lung disease, especially chronic obstructive pulmonary disease (COPD), personal history of cancer, family history of lung cancer, education, and asbestos exposure. Among the better-performing models, asbestos exposure is included only in the Liverpool Lung Project (LLP) and Bach models. No other occupational or environmental exposures have been included in the risk prediction models.

Ten Haaf [20] applied nine established lung cancer risk prediction models to the large data sets of the NLST and the PLCO and compared results to the risk factor eligibility criteria used in the NLST, using 5- and 6-year lung cancer incidence and mortality as outcomes. The specificity of the risk prediction models versus the NLST criteria were very similar (~62.2% to 62.6%), but four models had substantially higher levels of sensitivity (>78%) compared to that of the NLST criteria (71.4%) with respect to lung cancer incidence. The contrast in sensitivity for lung cancer mortality between the NLST criteria and the model predictions was even greater: 73.5% for the NLST versus 85.2% for the PLCO_m2012_ model and 83.8% for the TSCE (Two-Stage Clonal Expansion) and Bach models [20].

As part of its evidence review published in 2021, the USPSTF considered the merits and limitations of using a risk prediction versus risk factor (age and smoking only) approach to lung cancer screening. They compared the results of three risk prediction models (PLCO_m2012_, Lung Cancer Death Risk Assessment Tool (LCDRAT), and the Kovalchik model) with two sets of risk factor criteria, those of the NLST and the USPSTF 2013 criteria. In this comparison, only age, sex, and smoking covariates were included (Jonas 2021). They found that the risk prediction models led to a greater reduction in lung cancer deaths, a lower number of people needed to screen to prevent one lung cancer death, and a decrease in false positive tests compared to the NLST or USPSTF criteria [19]. The USPSTF acknowledges the potential improvements that the use of risk prediction models might bring to screening. However, in revising their recommendation for lung cancer screening in 2021, the USPSTF rejected the application of the risk prediction models in favor of retaining age, smoking pack-years, and years since smoking cessation as the sole criteria for recommending eligibility for LDCT. They cited a lack of direct empirical evidence (i.e., prospective clinical studies) in applying risk prediction models and possible barriers to implementation of the more complex eligibility criteria entailed in the use of the risk prediction models.

As noted above, the reception to the use of lung cancer risk prediction models in the implementation of lung cancer screening programs has been more favorable in Europe [17,18]. Favored models include the PLCO_m2012_ and the LLP_v2_ models [17,21]. The LLP_v2_ was used in the United Kingdom Lung Screening Trial (UKLS), a pilot randomized clinical trial [22]. The success of the UKLS has led to the implementation of lung cancer screening programs by the UK National Health Service initiated in 2019 in several regions of the United Kingdom, using the PLCO_m2012_ (adapted to the ethnic composition of the UK) and the LLP_v2_ to determine eligibility for LDCT among participants in the overall lung health program [18,23].

Results from one of the UK pilot studies, the Manchester Lung Health Check (LHC), have recently been published [24]. PLCO_m2012_ employing a 6 year lung cancer risk of ≥1.51% was used to select 1429 LDCT participants from a larger population for two rounds of screening, during which 62 lung cancers were detected. The authors applied the NLST eligibility criteria and the LLP_v2_ model (using ≥2.5% and ≥5% predicted lung cancer risk thresholds) to assess relative performance in the screened population. The use of these alternative selection criteria failed to detect 26% (LLP_v2_, ≥5% risk threshold), 18% (NLST), and 7% (LLP_v2_ ≥ 2.5% risk threshold) of the lung cancers that had been detected by the use of PLCO_m2012_.The authors also found that the PLCO_m2012_ model very likely underestimated lung cancer incidence in the study population, which had a high rate of co-morbidities. They conclude that further LDCT implementation requires the comparison of different risk prediction models to determine their relative performance in prospective studies.

### 2.4. Inclusion of Asbestos in Lung Cancer Risk Prediction Models

Three of the established lung cancer risk prediction models include asbestos exposure as a risk factor: the LLP_v2,_ Bach, and Spitz models. All treat asbestos exposure as a dichotomous variable (yes/no).

The Liverpool Lung Project risk prediction model was developed from a population-based case control study of lung cancer in Liverpool, England [25]. The study questionnaire included a lifetime occupational history, which listed jobs that were subsequently assessed by experts for likelihood, frequency, and intensity of asbestos exposure. This information was reduced to a dichotomous variable with asbestos exposure considered as “yes” if the person was exposed for at least 1 year during their working career. Details with respect to jobs, duration, frequency, and intensity regarding asbestos exposure are not provided. The lung cancer odds ratio for asbestos exposure in the final multivariate model was 1.89 (95% CI: 1.35–2.62), which was similar to the levels of risk individually associated with smoking for 1–20 years; prior personal history of cancer; and family history of lung cancer of early onset (<60 years) [25].

The LLP model was later validated in a prospective cohort study of the LLP, but few participants had asbestos exposures, rendering the validation inapplicable to the issue of asbestos exposure [26].

The current version of the LLP model, the LLP_v2_, includes asbestos exposure (yes vs. no) (“Can you recall any job or activity in which you were exposed to asbestos?”) [27]. This ascertainment of asbestos exposure was used in the UKLS (UK Lung Cancer Screening Trial) [28].

The UKLS CT screening data were also used to develop a different kind of model of lung cancer risk: a risk model that includes the finding of pulmonary nodules on baseline CT scan, the UKLS Nodule Risk Model. Unlike the previously discussed models, which address eligibility criteria, this model includes nodule interpretation and evaluates stratification of participants for follow-up. They found that a history of asbestos exposure per the LLP_v2_ model was useful in predicting which nodules represented lung cancer (OR = 1.8 (95% CI: 1.25–2.59) [29].

The Bach lung cancer risk prediction model was developed based on the Carotene and Retinol Efficacy Trial (CARET) [30]). The CARET study included ~4000 workers with a minimum of 5 years in a job with recognized occupational exposure to asbestos (5 years or work in a shipyard or selected construction trades). The model was later validated in a different cohort, though asbestos exposure was not addressed in the validation study [31].

An additional lung cancer risk model that includes a history of asbestos exposure developed by Spitz and colleagues was published in 2007. Asbestos exposure was defined as “employed within a documented asbestos-related occupation or industry” [32]. The model identified an odds ratio of 1.51 (95% CI: 1.09–2.08) for a history of asbestos exposure (yes/no).

### 2.5. Experience to Date with LDCT among Asbestos-Exposed Populations

#### 2.5.1. LDCT Screening of Asbestos-Exposed Populations

We searched MEDLINE, EMBASE, and the Web of Science through 31 December 2021 for relevant English language publications, using “asbestos”, ”low dose CT scan”, and “lung cancer screening” as key words. We obtained a total of 96 possible listings (with many duplicates among the databases) and reviewed abstracts to identify original studies that used low-dose chest CT scans to screen asbestos-exposed populations for lung cancer. We also reviewed the reference lists of identified studies. Conference abstracts generally contained insufficient information to be included. We did not conduct a full systematic review or meta-analysis, as these have been previously completed [33,34]. A total of 16 studies were identified: seven were excluded because they screened fewer than 150 people or used conventional dose CT scan for screening.

Nine non-randomized studies of asbestos-exposed populations with ≥150 participants reported results of LDCT screening for lung cancer between 2002 to 2019 [34,35,36,37,38,39,40,41,42,43,44]. Age, smoking history, and asbestos exposure were principal or exclusive eligibility criteria with frequent use of broader age and smoking ranges than were used in NLST or NELSON. Seven of these studies were included in a published systematic review and meta-analysis by Ollier et al. [33]. All the studies were included in a meta-analysis completed by Maisonneuve [34]. None of the studies used risk prediction models, NLST, or NELSON eligibility criteria to determine eligibility.

Combining the screening yield results of these nine studies yielded 86 lung cancers detected among 5548 ever smokers (1.55%) and 6 lung cancers detected among 1787 never smokers (0.33%). Comparison with NLST or the NELSON results is precluded by the heterogeneity of the study populations and the lack of sufficient detail on smoking history and age in the published studies.

Parameters of asbestos exposure varied widely among the nine studies by industry, occupation, duration of employment, presence or absence of pleural plaques, and others. One-half of published studies screened people only if they had a history ≥15 years of asbestos exposure, while one-half used a history of 1 or 5 years of significant asbestos exposure as a screening criterion. The studies with the highest lung cancer detection yields by LDCT also tended to have study populations with the highest prevalence (43% to 89%) of pleural plaques on low-dose CT scans [37,41,44]. The pleural plaques were generally identified by LDCT.

Only one study has reported on lung cancer mortality follow-up of application of LDCT among an asbestos-exposed population. Barbone and colleagues examined nine-year mortality follow-up of a non-randomized study of 926 asbestos-exposed workers who were enrolled in an asbestos surveillance program in Northeastern Italy, a major shipbuilding area. The group had undergone at least two periodic LDCT scans beginning in 2002 [38,45]. They were mostly men with a mean age of 58 years at study onset and a long history of exposure to asbestos (mean duration of 30 years); one-third never smoked, and median pack-years of cigarettes among smokers was 18.5. The comparison group of 1507 people had more smokers but a lower average level of asbestos exposure and underwent chest-rays (CXR). The LDCT study group showed a lung cancer SMR of 0.55 (95% CI: 0.24–1.09), and the CXR study group had a lung cancer SMR of 2.07 (95% CI: 1.53–2.71) in comparison with regional mortality rates. In a Cox proportional hazard analysis adjusting for age, smoking, and asbestos exposure level, the LDCT study group showed a hazard ratio for lung cancer of 0.41 (95% CI: 0.17–0.96) versus the CXR study group. Although differences in the lung cancer risk factor profile between the study groups and the non-random nature of the study likely accounted for some of the observed difference in lung cancer mortality, LDCT detection of lung cancer also likely played an important role in the lung cancer mortality reduction.

Loewen and colleagues used LDCT to study a population that mostly had environmental exposure to Libby amphibole asbestos in Libby Montana. The population was aged 50 to 84 years, had a >20 pack-year history of tobacco use, and had a history of asbestos-related pleural or pulmonary fibrosis on a previous high-resolution CT scan. Seventeen lung cancers were detected: 71% were Stage 1 (59%) or Stage 2 (11%) [46].

#### 2.5.2. LDCT Screening of Asbestos-Exposed Never Smokers

Kato et al. performed a one-time LDCT screening of 2132 asbestos-exposed workers, principally from shipbuilding, construction, and manufacturing sectors in Japan. Eligibility criteria included: (1) work in asbestos manufacturing for ≥1 year; or (2) work in other asbestos-exposed industries for >10 years; or (3) evidence of pleural plaques on CXR or chest CT scan. In total, 89% of participants had pleural plaques on CT scan. Details on the extent of smoking were not provided. They detected 3 lung cancers among 444 never smokers (0.7%) and 42 lung cancers among 1651 smokers (2.5%) [44].

Brims and colleagues in Australia screened 960 individuals with a history of exposure to asbestos, defined as having ≥3 months of occupational exposure to asbestos or radiographic evidence of pleural plaques [43]. Mean cigarette consumption in the study population was modest (17 pack-years). Only 3.6% of the cohort would have been eligible for lung cancer screening under the 2013 USPSTF eligibility guidelines. In total, 2 lung cancers were detected by LDCT among 325 never smokers, a screening yield of 0.62%. Additionally, 5 lung cancers were detected among 569 smokers, a screening yield of 0.88%. This study cohort was updated to include 1743 people overall, including 595 never smokers, though the number of never smokers who developed lung cancer was not reported [47].

#### 2.5.3. LDCT Screening of Other Occupational Populations at Risk of Lung Cancer

From 2000 to the present, Markowitz and colleagues have used low-dose chest CT scanning to screen ~14,000 workers who had worked at nuclear weapons facilities in 13 mostly non-metropolitan U.S. communities with variable exposure to asbestos, radiation, beryllium, nickel, chromium, and other toxins. Eligibility criteria included age (≥50 years), smoking, occupation (production, maintenance or laboratory worker), and, if present, asbestos-related radiographic parenchymal or pleural fibrosis and/or a positive beryllium lymphocyte proliferation test. In a 2018 report on 7189 of these workers, all of whom had a smoking history, the proportions with screen-detected lung cancer were 0.83% at baseline and 0.51% on annual scan. Stage distribution at diagnosis was favorable: of 80 detected lung cancers, 59% (*n* = 47) were Stage 1, and 10% (*n* = 8) were Stage 2. Study strengths included high study compliance; high credibility with the study population through labor union co-sponsorship; implementation in community settings; excellent follow-up; and use of a standardized protocol with demonstrated quality [48].

To delineate the occupational contribution to aggregate lung cancer risk, Markowitz et al. compared two sub-groups of the study population: Group A met NLST eligibility criteria for age (≥55 years) and smoking history (≥30 pack-years); Group B did not meet these NLST study criteria but met National Comprehensive Care Network (NCCN) Group II criteria (age ≥ 50 years, 20–29 pack-year smoking history, and occupational risk). Both groups had occupational exposures. Group B had a lower overall lung cancer risk compared to the NLST study based on age and smoking profile but had occupational risk of lung cancer. The lung cancer screening yield of Group A (1.5%, 95% CI: 0.88–2.12%) was similar to that of Group B (1.36%, 95% CI: 0.85–1.87%). Both were similar to the screening yield of the original NLST study (1.0%, 95% CI: 0.88–1.12%). These results indicate that, in the presence of occupational risk, younger people with lesser smoking histories could nonetheless benefit from LDTC screening [48].

Although the Markowitz et al. study did not include mortality follow-up, the shift in diagnosis of earlier stage lung cancers with LDCT screening is consistent with the NLST and NELSON trials that demonstrated favorable mortality reductions [48].

A second study of LDCT screening among US nuclear weapons workers—construction workers—was completed by Welch, Dement, and others [49]. The study group had a lower age and smoking threshold than the NLST study criteria (age ≥ 50 years and smoking ≥ 20 pack-years), but they had additional lung cancer risk, as defined by 5 years of work in the construction industry (exposure to asbestos, silica, beryllium, chromium, radiation, or welding), evidence of radiographic asbestosis or pleural plaques, or spirometric evidence of chronic obstructive pulmonary disease. They found a favorable stage distribution among CT-detected cancers: 20 of 30 (67%) detected lung cancers were Stage 1 (57%) or Stage 2 (10%) disease. As in the Markowitz et al. study, the lung cancer screening yield at baseline scan was 1.7% (21 of 1260 participants), a result that was similar to the NLST results, despite the fact that less than one-half of participants met the NLST eligibility criteria [49]).

Dement and colleagues used this nuclear weapons construction worker cohort and a related larger construction worker population to develop a lung cancer risk prediction model (BTMed model) that includes age, gender, race, smoking history, spirometry, chest X-ray finding of parenchymal fibrosis and/or pleural plaques, occupational history of ≥5 years of work in construction, body mass index, and personal history of cancer [50]. Applying the lung cancer screening criteria described above in the Welch et al. study yielded an 85.6% sensitivity, a 56.8% specificity, and a 4.2% positive predictive value. This level of sensitivity compares favorably with that of the PLCO_m2012_ model (85.2%) and of the Two-Stage Clonal Expansion (83.8%), though the specificity is somewhat lower. Dement et al. applied the 2013 USPSTF-recommended screening criteria (age 50–80 years, ≥30 pack-years of smoking, and quitting <15 years in past) to their dataset and obtained a 50.9% sensitivity, an 81.2% specificity, and a 5.7% positive predictive value [50]. This large decline in sensitivity, from 85.6% to 50.9%, using the different eligibility criteria, represents a failure of the 2013 USPSTF criteria (which exclude occupation) to detect as many as 40% of the lung cancers detected in the study.

## 3. Current Challenges to Application of LDCT among AEP

### 3.1. Setting LDCT Eligibility Criteria for Asbestos-Exposed Populations

Few attempts have been made to establish a recommended set of exposure criteria, alone or in combination with other risk factors, for decision making with respect to the use of LDCT in asbestos-exposed populations. The few risk prediction models that include asbestos exposure as a risk factor (LLP_v2_, Bach, and Spitz models) have not been applied specifically to asbestos-exposed populations and have not included sufficient numbers of asbestos-exposed people to date to provide useful conclusions. The new LDCT lung cancer screening implementation program undertaken by the UK National Health Service, which uses LLP_v2_, may provide some useful data on this issue. The BTMed model developed by Dement and colleagues includes construction occupations as a variable in the model, but this variable is not specific to asbestos, since construction workers have multiple exposure to lung carcinogens, including asbestos, silica, diesel exhaust, and chromium.

In 2014, a group of international experts in asbestos-related diseases met in Helsinki and recommended that the lung cancer risk level associated with the NLST study population be used as the threshold risk level in organized low dose CT scan programs for screening asbestos-exposed workers for lung cancer [51]. The Markowitz and Welch studies summarized above approximated this approach by including workers who were younger and had a lesser smoking history (and therefore had lower lung cancer risk) than the NLST study population in combination with occupational exposures (which raised the lung cancer risk). These studies were not specifically of asbestos-exposed populations. The Helsinki recommendation was made before completion of the NELSON clinical trial, numerous modeling studies, and revision of the USPSTF eligibility guidelines in 2021 and therefore requires re-examination. In addition, its support for the inclusion of LDCT in medical screening of AEPs only as part of well-organized programs fails to recognize the substantial progress in LDCT research in the past 8 years and the universal turn towards the implementation of LDCT for high-risk individuals.

In 2017, an expert working group in France made recommendations for the application of LDCT for lung cancer screening for workers who have a history of exposure to Group 1 IARC occupational lung carcinogens, including asbestos [52]. They conducted a scientific literature review and developed an expert consensus opinion to identify the magnitude of lung cancer risks associated with these Group 1 carcinogens, alone and in combination with cigarette smoking, and identified the combinations that equaled or exceeded the lung cancer risk associated with NLST eligibility criteria. They assumed a multiplicative joint effect between the occupational carcinogenic agent and tobacco in estimating the relative risks. They estimated that the relative risk of lung cancer associated with the NLST study eligibility criteria (≥30 pack-year history of cigarette smoking) was 30. Using the target relative risk level of 30, exposure to each of the IARC Group 1 lung carcinogens in combination with 20–29 pack years of smoking met or exceeded the target risk level.

The French expert working group developed more detailed recommendations regarding asbestos exposure. They defined “high” asbestos exposure as having worked (1) continuously for 1 year in asbestos-product manufacturing, in shipyards, or in selected construction trades, or (2) periodically for 10 years as mechanics, brake workers, or cutting asbestos cement. Intermediate exposure was defined as all other occupational exposures that involve disturbance of asbestos-containing materials. All workers with intermediate or high levels of exposure to asbestos who smoked ≥20 pack-years of cigarettes (including former smokers who had who quit ≤15 years previously) were assigned a relative risk of lung cancer of 30 or greater, indicating that their risk matched or exceeded the study group in the NLST trial. Never smokers who were occupationally exposed to asbestos at any level did not reach a relative risk of 20, per the Delva et al. analysis [52].

The French recommendations proposed that a screening study be conducted among workers whose eligibility is based on age, smoking, and exposure to IARC Group 1 lung carcinogens. Indeed, Delva and co-investigators have begun such a study in numerous regions in France [53]).

Since the publication of this set of French recommendations in 2017, the results of the NELSON trial and modeling studies led the USPSTF to lower the age and smoking levels for eligibility for LDCT-based lung cancer screening (≥age 50 years and ≥20 pack-years). The associated estimated relative risk level of lung cancer in the French analysis would be 20. Accordingly, workers with intermediate asbestos exposure ≥10 years and all workers with high asbestos exposure would be recommended for lung cancer screening even if their smoking history was less than 20 pack-years.

What do we do now? Perhaps the use of the LLP_v2_ model in the UK Lung Cancer Screening Program in the general population will shed light on the combination of lung cancer risk factors (including asbestos) that optimize lung cancer detection and can be generalized to other populations. Other current limited studies that are focused on asbestos-exposed groups may also provide generalizable results even if they are not tests of risk prediction models.

Interim practical recommendations are needed in order to permit workers at high risk of lung cancer to benefit from the remarkable tool represented by LDCT-based screening. Given current knowledge, it is reasonable to offer LDCT screening for lung cancer to workers aged ≥ 50 years who have a history of 5 or more years of asbestos exposure (beginning 20 or more years previously), who have or have had a smoking history of at least 10 pack-years, and, for former smokers, with no limit on years since quitting [54,55]. Note that this level of smoking-related risk is, on average, less than that associated with the current USPSTF-defined smoking history (≥20 pack-years and <15 years since quitting) but is compensated by the added risk of lung cancer associated with asbestos exposure. Workers with less than 5 years of asbestos exposure but whose exposure was especially intense should also be considered for LDCT screening. For asbestos-exposed workers who never smoked but who have one or more other lung cancer risk factors, including asbestos-related parenchymal or pleural fibrosis; chronic obstructive or interstitial lung disease; a family history of lung cancer; a personal history of cancer; or exposure to multiple occupational lung carcinogens, offering LDCT should also be strongly considered [56,57]. Whether LDCT screening should be offered to workers exposed to asbestos but who have never smoked and have no other risk factors depends on the threshold level of lung cancer risk at which LDCT screening is justified, an area of evolving research and consensus [58,59,60] Kerperl-Fronius 2022; ten Haaf 2022; Tammemägi 2014).

### 3.2. Identifying at Risk Asbestos-Exposed Populations

Numerous countries have regulations, registries, or surveillance programs that mandate, enroll, or conduct periodic medical screening for AEPs. The radiologic component of mandated asbestos examinations was summarized for six European, two North American, and two Asian countries in the 2014 Helsinki Criteria update on Asbestos [61]. As of the 2014 Helsinki update, the only country to offer low-dose CT scans for lung cancer screening was Germany, which offered low-dose CT scans for high-risk workers (defined as first exposure before 1985; ≥10 years of asbestos exposure; ≥30 pack-years tobacco smoking history; ≥55 years of age; no history of lung cancer; and able to undergo chest surgery). It is not clear how many countries offer medical surveillance after job termination or retirement. The US, for example, only requires employers to offer asbestos-based medical examinations to current employees, which end at job termination.

Italy has had a national exposure surveillance system for occupational carcinogens, including asbestos, and regional activities that are dedicated to health screening of former asbestos-exposed workers for many years and has been guided in recent decades by national consensus conferences attended by relevant experts [62,63,64]. These programs have been sponsored by occupational health services connected to regional health service departments and utilize numerous cohort studies previously conducted among workforces of large asbestos-using facilities. Such a regulatory, organizational, and informational framework would appear to be an excellent infrastructure on which to build an LDCT-based lung cancer screening program.

In France, national regulations mandate post-occupational medical surveillance [52]. In a demonstration of how such surveillance can be used to conduct LDCT-based screening, Delva and colleagues are conducting a multifaceted pilot study of LDCT screening of workers exposed to occupational lung carcinogens in two specialized centers in France. They published a protocol of this feasibility study in 2019 [53] and based their rationale of risk eligibility on a previous expert-driven consensus process. They defined high-risk workers as those aged 55 through 74, who have various combinations of smoking pack-years and cumulative exposure to occupational lung carcinogens. Asbestos-exposed workers from high-risk settings (e.g., the manufacture of asbestos-containing materials, shipbuilding, or fireproofing) are eligible if they have worked in those industries for at least 5 years and have smoked at least 20 pack-years. People who quit smoking 15 or more years ago or who have never smoked are excluded from the study. Subjects are recruited through an invitation letter and triage questionnaire sent to the general population aged 55 through 74 years. Based on the results of the questionnaire regarding occupational history and smoking history, prospective participants are invited to a regional occupational health center for final eligibility determination. Eligible people undergo spirometry, LDCT for two years (baseline and two annual scans), and, if needed, smoking cessation counseling.

The Delva et al. study goals and endpoints are comprehensive, and results should be instructive [53]. They include study feasibility, study participation and retention, smoking cessation counseling impact, utility of risk determination tools, medical and social impact of screening, and cost effectiveness. Notably, the lung cancer risk threshold associated with the Delva et al. eligibility guidelines exceeds that of the current recommended USPSTF eligibility criteria (ages 50–80 years; ≥20 pack-years; and ≤15 years since quitting). However, this should not undermine important insights that the Delva et al. study may yield.

Other high-income countries have well-established medical surveillance programs for asbestos-exposed workers. Japan mandates periodic medical examinations for asbestos-exposed individuals under its Act on Asbestos Health Damage Relief passed in 2006 [65].

For five decades, Germany has had a mandatory registration and surveillance system for workers with a history of asbestos exposure; previous screening has employed high-resolution CT scans for the detection of asbestos-related diseases [66].

The United States has very few organized programs that track formerly asbestos-exposed workers or offer medical surveillance to asbestos-exposed workers, especially post-retirement when lung cancer risk is likely to be highest. The Department of Energy Former Worker Medical Screening Program is described above [48,49,50]. The US Navy has for several decades sponsored the Navy Asbestos Medical Surveillance Program [67], though few results appear to have been published in recent decades [68]. This program is offered to active service personnel and likely excludes personnel formerly exposed to asbestos in the Navy who are at highest risk: retirees aged 50 and over. The Federal (U.S.) Occupational Safety and Health Administration only requires health surveillance of current employees with asbestos exposure, thereby excluding retirees. Very few labor unions offer asbestos-based medical screening to their members, and employers neither track nor provide any asbestos-based services to retirees. Occupational health clinics offer asbestos medical surveillance to individual patients, but these are not frequently undertaken in organized and funded screening programs.

### 3.3. Modifying Health Care Information Systems and Health Provider Practice to Facilitate Identification of Asbestos-Exposed Patients for LDCT Screening

Although challenging, integrating the assessment of occupational asbestos exposure and lung cancer risk into primary care encounters and clinical decision-making offers the opportunity to reach a much larger population than mandated health examinations targeting selected industries or occupations. Given the widespread use of asbestos in past decades in high-income countries, such penetration, if possible, of primary health care systems would be a useful development.

Electronic health record (EHR) systems can facilitate the collection of information and support and encourage clinical decision making that includes occupational health concerns [69,70]. Some key limitations to healthcare provider acceptance of this integration in the US arethe lack of time to address occupational aspects of health in the brief clinical encounters that increasingly typify the practice of medicine and clinicians’ perception that impacting working conditions is beyond their control [71].

The use of EHR systems can overcome or reduce these barriers to decision making about whether an individual patient might benefit from LDCT for lung cancer screening. EHR systems presumably already obtain and utilize age and smoking history information to assist in deciding who should receive LDCT-based screening. In the case of asbestos, usual (or longest) job title held, industry, and calendar years worked are likely to permit the identification of a large proportion of individuals who had asbestos exposure during their working careers, especially among people aged 50 and over. This reasonably straightforward information could be collected as part of the EHR, and simple algorithms that combine age, smoking history, and occupation could be used to indicate to the clinician whether the patient is a candidate for LDCT-based lung cancer screening. Indeed, it is possible to test whether use of combinations of broad occupational categories in combination with industrial categories (e.g., skilled trades in construction or installation, maintenance, and repair occupations in manufacturing) and calendar years worked might provide adequate information about probable asbestos exposure in people at selected age thresholds to assist in LDCT decision making.

### 3.4. Educating and Motivating Asbestos-Exposed Populations to Participate in Annual LDCT Screening

Despite the fact that LDCT for lung cancer screening has been approved for use and reimbursement in the United States since 2013, uptake in the general population has been slow. Kee et al. used self-reported survey data from a national survey, the Behavioral Risk Factor Surveillance Survey, conducted in 2018, and estimated that 17.7% of people eligible for LDCT according to the 2013 USPSTF recommended criteria based on age and smoking alone participated in annual LDCT screening for lung cancer [72].

Myriad reasons underlie the reluctance of high-risk individuals to undergo annual LDCT screening, including fear of lung cancer, fatalistic attitude towards the diagnosis of lung cancer, stigma attached with smoking (i.e., self-blame), concern about radiation, among others. To date, research on lung cancer screening hesitancy among blue-collar workers has been limited.

Ali and colleagues evaluated the high-risk group that declined to participate in the United Kingdom Lung Screening Trial (UKLS) and found that females, current smokers, people over age 70, and people in the lowest two quintiles of socioeconomic status were substantially more likely to decline to participate in the trial [73]. Among the identified emotional barriers, fear of lung cancer and avoidance of lung cancer were primary obstacles to screening. Yousaf-Kahn et al. used the population of the NELSON trial in Holland and compared people eligible for this study who decided not to participate with the study control group. They found that current smokers and people with a lower educational level were less likely to participate in the study; these results were similar to those of the UKLS [74].

Despite publication of the NLST results one decade ago and recommendation of LDCT by the USPSTF 9 years ago, little attempt has been made in the US to inform and motivate the general population about this life-saving screening method. A major public health campaign is needed in addition to targeted implementation programs (as is occurring, for example, in the UK and France) to order to accelerate the uptake of annual LDCT and disseminate the benefits of its use in the US. Identifying how best to reach and convince blue collar workers, including asbestos-exposed populations, to participate in LDCT screening should be an important part of such initiatives.

## 4. Conclusions

Over 70 years of research has demonstrated that occupational asbestos exposure is an important cause of lung cancer mortality. Lung cancer deaths occur today as a result of widespread exposure in past decades and will persist due to continuing exposure to asbestos in many countries. Annual low-dose CT scanning reduces lung cancer mortality in the general population, as conclusively shown in studies that focus on age and smoking as the chief determinants of lung cancer risk. Indeed, age and smoking remain the foundational risk factors for lung cancer screening at present in the United States. Risk prediction models developed from large studies of lung cancer offer the opportunity to include additional risk factors such as asbestos exposure to define eligible populations, but their field implementation has only recently begun.

The use of LDCT to date in asbestos-exposed populations has shown findings consistent with the results in the NLST and NELSON trials, but studies have mostly been modest in size, variable in design, and short term in follow-up. Given the current state of LDCT research, whether large controlled studies of LDCT among asbestos-exposed populations are necessary, feasible, or ethical is questionable. More pertinent questions revolve around how best to implement LDCT among asbestos-exposed workers, including how to identify such workers, to organize and fund screening programs, to inform and motivate people about screening, and to influence health care practice to increase recognition of occupational lung cancer risk factors in decision making.

Screening workers aged 50 years and older with a history of 5 or more years of exposure to asbestos in combination with either (a) a history of smoking (including those who smoked ≥10 pack-years and those who are >15 years since quitting), or (b) other lung cancer risk factors (asbestos-related fibrosis; chronic obstructive or interstitial lung disease; a family history of lung cancer; personal history of cancer; or exposure to multiple occupational lung carcinogens) is a sensible approach, given current knowledge. Workers with less than 5 years of asbestos exposure but whose exposure was especially intense should also be considered for LDCT screening. Whether LDCT screening of workers exposed to asbestos but who never smoked and have no other lung cancer risk factors is justified is uncertain and requires additional research and analysis.

LDCT-based screening is an unprecedented opportunity to intervene in the natural history of a lethal occupational disease. Its promotion and use should be an urgent priority.

## Data Availability

Not applicable.
